# Automated Risk Stratification for Corrected QT Interval (QTc) Prolongation Related to Haloperidol Use

**DOI:** 10.7759/cureus.117922

**Published:** 2026-10-14

**Authors:** Erwin Ni, C. William Heise

**Affiliations:** 1 Emergency Medicine, University of Arizona College of Medicine, Phoenix, USA; 2 Medical Toxicology, Banner - University Medical Center, Phoenix, USA

**Keywords:** clinical risk score, drug-induced qtc prolongation, drug-induced torsades de pointes, haloperidol, haloperidol-induced

## Abstract

Objectives: Automated clinical risk scores have been developed to identify patients at high risk of medication-induced corrected QT interval (QTc) prolongation and torsades de pointes (TdP). This study aimed to describe the incidence and magnitude of QTc prolongation following haloperidol administration among hospitalized patients identified as high risk for TdP by an automated, modified Tisdale risk score. A secondary objective was to identify clinical and medication-related factors associated with marked QTc prolongation in this high-risk population.

Methods: This was a cross-sectional observational study approved by the Institutional Review Board. We included 36 patients aged >18 years admitted to one of 15 hospitals in Arizona or neighboring states between January 2020 and February 2026. Patients received at least one dose of haloperidol during their stay. Outcomes investigated were a relative QTc prolongation of ≥20 ms above baseline, or an absolute QTc ≥500 ms, as defined using Fridericia’s formula. Patient demographic information, laboratory values, and medication exposures were obtained from the chart to determine risk factors for QTc prolongation.

Results: Eighteen (50%) patients given haloperidol had a relative QTc increase of ≥20 ms, and nine (25%) had an absolute QTc of ≥500 ms. Concurrent diuretic use was significantly associated (p = 0.014) with absolute QTc prolongation of ≥500 ms.

Conclusions: There is a significant incidence of both relative and absolute QTc prolongation in patients identified as high risk by an automated clinical risk score who were prescribed haloperidol. These findings support further investigation of automated risk stratification as a means of identifying patients who may warrant targeted QTc monitoring and medication review. Caution may be advised with the concurrent use of haloperidol and diuretics in identified high-risk patients.

## Introduction

Torsades de pointes (TdP) is a potentially lethal polymorphic ventricular tachycardia often associated with medication-related corrected QT interval (QTc) prolongation. A QTc of over 450 ms in male and 460 ms in female patients is considered prolonged [1]. The risk of TdP increases two- to three-fold with a QTc of more than 500 ms [2]. TdP can degenerate into ventricular fibrillation, a heart rhythm incompatible with life and requiring emergent defibrillation. Many medications are associated with QTc prolongation and TdP, including but not limited to antiarrhythmics, antipsychotics, diuretics, antiemetics, antifungals, and several classes of antibiotics. Risk factors for QTc prolongation and TdP include advanced age, female sex, bradycardia, preexisting heart disease, and underlying electrolyte disturbances (hypokalemia, hypocalcemia, and hypomagnesemia) [1]. Models have been created to help quantitatively estimate the risk of TdP in hospitalized patients receiving a potentially QTc-prolonging medication. These models compile common patient data elements, such as serum electrolyte levels and a history of heart disease, into a risk score to predict the risk of QTc prolongation and TdP [3]. Tisdale et al. conceptualized one such risk score, with weighting of various factors based on relative risk [4]. These models can be used to identify patients at high risk of QTc prolongation and TdP.

The rising prevalence of psychiatric disorders has led to greatly increased antipsychotic medication use [5]. Several antipsychotic medications have been associated with QTc prolongation and TdP. Pimozide, sertindole, thioridazine, droperidol, and haloperidol have all been documented to cause QTc prolongation, TdP, and sudden death [6]. While the precise mechanism of how various antipsychotics prolong the QTc and induce TdP is not fully known, it is thought that the inhibition of cardiac myocyte potassium rectifier channels, particularly the hERG channels, plays a major role [5,6]. The inhibition of these potassium channels prolongs ventricular cardiac myocyte repolarization, which increases the QT interval [7].

There is mixed evidence regarding the risk of QTc prolongation and TdP in haloperidol specifically. Haloperidol is a typical antipsychotic commonly used in the hospital or emergency department to control agitation. At lower doses, it may also be used to manage nausea and vomiting. Haloperidol is associated with QTc prolongation, and cases of TdP have been reported with haloperidol use [6,8]. However, haloperidol’s apparent risk profile may be confounded by the fact that it is typically used in acutely ill hospitalized patients, who often have multiple comorbidities and may be taking other medications that can cause QTc prolongation and TdP [9]. Some studies suggest that haloperidol may be relatively safe at low dosages [10]. Given these mixed findings, along with a relative dearth of literature directly investigating any dose-dependent arrhythmogenic effects of haloperidol, it would be clinically useful to conduct a study investigating these questions in the hospital setting.

Automated implementation of risk scores may help identify hospitalized patients with multiple concurrent risk factors for drug-induced QTc prolongation. However, the frequency and magnitude of QTc changes following haloperidol administration among patients identified as high risk by such systems remain incompletely characterized. The objective of this study was therefore to describe QTc changes following haloperidol administration in hospitalized patients identified as high risk for TdP using an automated risk score. We additionally examined demographic, clinical, laboratory, and medication-related factors associated with QTc prolongation.

## Materials and methods

This was a cross-sectional chart review using inpatient data from a large healthcare system in Arizona and nearby states. Patients were admitted to one of 15 hospitals from January 1, 2020, to February 1, 2026. Eligible patients were ≥18 years of age, received at least one dose of haloperidol (intravenous, intramuscular, or oral) during their hospital stay, and were flagged for increased TdP risk with a modified Tisdale TdP risk score >11. The modified Tisdale score differs from the original validated Tisdale score in that 4 points were assigned if the most recent electrocardiogram (ECG) recorded a QTc >500 ms, whereas the standard Tisdale score assigns 2 points if the admission QTc >450 ms [11]. Electronic medical records were queried to extract demographic data (age, sex), comorbidities (including structural heart disease, heart failure, and renal disease), laboratory values (serum potassium, magnesium, and calcium), medication exposure (24-hour cumulative haloperidol dose prior to ECG, route, and concurrent QTc-prolonging medications), and clinical variables such as TdP risk score.

QTc-prolonging medications were identified based on the CredibleMeds classification [12] of drugs with a known risk of TdP, as described by Gallo et al. Medications included antibiotics (azithromycin, levofloxacin, ciprofloxacin, erythromycin, moxifloxacin, and clarithromycin); antiemetics (ondansetron); antiarrhythmics (amiodarone, sotalol, flecainide, dofetilide, dronedarone, ibutilide, quinidine, and procainamide); other medications (propofol, escitalopram, citalopram, donepezil, methadone, hydroxychloroquine, cilostazol, and oxaliplatin); antipsychotics (haloperidol and chlorpromazine); and antifungals (fluconazole, voriconazole, posaconazole, pentamidine, and itraconazole).

We excluded patients with paced rhythms from the study. Patients were required to have a baseline 12-lead ECG or cardiac telemetry study obtained prior to haloperidol administration and at least one follow-up ECG or cardiac telemetry study obtained within 24 hours after administration. Patients without an acceptable baseline or follow-up ECG or telemetry study were excluded. The most recent ECG or telemetry study prior to any haloperidol administration was used to calculate a baseline QTc. Cardiac telemetry QTc monitoring has been shown to be effective and highly correlated with ECG QTc values during the COVID-19 pandemic [13]. QTc intervals were calculated using Fridericia’s formula for both ECG and telemetry data. The primary outcome was the prevalence of an increase in QTc ≥20 ms from baseline following haloperidol administration. Secondary outcomes included the occurrence of marked QTc prolongation (QTc ≥500 ms) and documented ventricular arrhythmias.

Descriptive statistics were used to summarize patient characteristics and QTc changes. Continuous variables were reported as mean ± standard deviation or median with range if more appropriate. Categorical variables were reported as counts and percentages. The chi-square test and Student’s t-test were used to compare groups and identify potential risk factors for QTc prolongation. A two-sided p value of <0.05 was considered statistically significant. Analyses were conducted using Microsoft Excel (Microsoft Corporation, Redmond, WA).

## Results

Overall patient characteristics

A total of 108 hospitalized patients were evaluated for inclusion. Of these, 72 patients were not eligible due to exclusion criteria, primarily for not having timely ECG data. The final data set consisted of 36 patients with a mean age of 71.2 ± 15.4 years and a mean TdP risk score of 13.47 ± 1.42. Demographic data, laboratory values, and comorbidities of the study population are shown in Table 1.

**Table 1:** Characteristics of study population TdP, torsades de pointes; GFR, glomerular filtration rate; CHF, congestive heart failure; CAD, coronary artery disease; MI, myocardial infarction

Patient characteristic	Overall
Number of patients	36
Age (years)	71.2 ± 15.4
Female sex	23 (64%)
Heart rate (bpm)	84.9 ± 22.4
TdP risk score	13.47 ± 1.42
Diuretic use	24 (67%)
Blood chemistry	
K (mmol/L)	3.75 ± 0.61
Mg (mmol/L)	1.96 ± 0.25
Ca (mmol/L)	8.59 ± 0.83
GFR (mL/minute/1.73 m^2^)	69.1 ± 26.0
CHF	19 (53%)
History of CAD or MI	18 (50%)
Aortic stenosis	4 (11%)
Median haloperidol dose (mg)	2.5 (1-40)
Patients receiving ≥5mg haloperidol	15 (42%)

Risk factors for QTc prolongation

Overall, 18 (50%) patients had a relative QTc increase of more than 20 ms (Table 2), and nine (25%) had an absolute QTc greater than 500 ms (Table 3). Age, TdP risk score, and gender distribution were comparable between patients in all comparison groups. Preexisting comorbidities, GFR, and heart rate showed no significant differences between groups. Diuretic use was significantly associated with an absolute QTc ≥500 ms.

**Table 2:** Patient characteristics of groups with relative QTc prolongation (≥20 ms) TdP, torsades de pointes; GFR, glomerular filtration rate; CHF, congestive heart failure; CAD, coronary artery disease; MI, myocardial infarction; QTc, corrected QT interval

Patient characteristic	QTc not increased	QTc increased (≥20 ms)	Test statistic	p value
Number of patients	18 (50%)	18 (50%)	-	-
Age (years)	70.4 ± 16.9	72.0 ± 14.3	t = 0.298	0.768
Female sex	13 (72%)	10 (56%)	χ² = 1.084	0.298
Heart rate (bpm)	92.0 ± 23.0	77.8 ± 19.8	t = -1.987	0.055
TdP risk score	13.28 ± 1.36	13.67 ± 1.50	t = 0.815	0.421
Diuretic use	10 (56%)	14 (78%)	χ² = 2.000	0.157
Blood chemistry				
K (mmol/L)	3.77 ± 0.53	3.74 ± 0.70	t = -0.178	0.861
Mg (mmol/L)	1.95 ± 0.18	1.96 ± 0.31	t = 0.077	0.939
Ca (mmol/L)	8.76 ± 0.85	8.43 ± 0.79	t = -1.196	0.240
GFR (mL/minute/1.73 m^2^)	63.7 ± 28.9	74.4 ± 22.3	t = 1.238	0.224
CHF	9 (50%)	10 (56%)	χ² = 0.112	0.738
History of CAD or MI	9 (50%)	9 (50%)	χ² = 0.000	1.000
Aortic stenosis	1 (6%)	3 (17%)	χ² = 1.125	0.289

**Table 3:** Patient characteristics of group with marked (≥500 ms) absolute QTc prolongation TdP, torsades de pointes; GFR, glomerular filtration rate; CHF, congestive heart failure; CAD, coronary artery disease; MI, myocardial infarction; QTc, corrected QT interval ^*^Statistically significant (p < 0.05)

Patient characteristic	QTc <500 ms	QTc ≥500 ms	Test statistic	p value
Number of patients	27 (75%)	9 (25%)	-	-
Age (years)	71.8 ± 15.2	69.5 ± 16.7	t = -0.384	0.703
Female sex	19 (70%)	4 (44%)	χ² = 1.967	0.161
Heart rate (bpm)	88.9 ± 22.9	72.9 ± 16.3	t = -1.931	0.062
TdP risk score	13.30 ± 1.38	14.00 ± 1.50	t = 1.296	0.204
Diuretic use	15 (56%)	9 (100%)	χ² = 6.000	0.014^*^
Blood chemistry				
K (mmol/L)	3.71 ± 0.65	3.89 ± 0.44	t = 0.735	0.490
Mg (mmol/L)	1.90 ± 0.21	2.20 ± 0.27	t = 3.416	0.013^*^
Ca (mmol/L)	8.53 ± 0.77	8.79 ± 0.99	t = 0.822	0.417
GFR (mL/minute/1.73 m^2^)	68.4 ± 25.8	71.1 ± 28.1	t = 0.270	0.789
CHF	12 (44%)	7 (78%)	χ² = 3.009	0.083
History of CAD or MI	12 (44%)	6 (67%)	χ² = 1.333	0.248
Aortic stenosis	3 (11%)	1 (11%)	χ² = 0.000	1.000

The median cumulative dose of haloperidol given in the 24 hours prior to ECG was 2.5 mg (1-40 mg). Fifteen (42%) patients received a dose of haloperidol equal to or greater than 5 mg in the 24 hours prior to QTc measurement. Differences in the magnitude of relative QTc prolongation are shown in Table 4.

**Table 4:** Relative QTc prolongation (ms) by haloperidol dose QTc, corrected QT interval

Patient characteristic	<5 mg	≥5 mg	Test statistic	p value
QTc prolongation	6.1 ± 59.2	40.1 ± 41.7	t = 1.907	0.065

## Discussion

The primary results of this study were that half of high-risk hospitalized patients who received haloperidol had a clinically significant QTc prolongation of 20 ms or more, and a quarter of the study population had a QTc of more than 500 ms, which is associated with a two- to three-fold increase in the incidence of TdP. This is a relatively high incidence of both absolute and relative QTc prolongation, and these results suggest that an automated risk score may be a useful method to identify high-risk patients for QTc prolongation and TdP prior to prescribing potentially QTc-prolonging medications.

Several formulas have previously been used to normalize the QT interval according to the heart rate. Bazett’s formula was historically the most popular and is often still used in automated QTc measurements today. However, Bazett’s formula may overcorrect the QT interval when the heart rate is very tachycardic or bradycardic, leading to an overestimation of the incidence of QTc prolongation [14,15]. Fridericia’s formula was chosen over Bazett’s formula since it produces more reliable results when the heart rate is significantly elevated, as is often the case in acutely ill hospitalized patients.

When analyzing the effects of QTc-prolonging medications, it is important to consider both the relative prolongation of QTc from baseline, as well as the absolute value of the QTc. Generally, adverse outcomes such as TdP are associated with a prolonged absolute QTc, especially over 500 ms. If a patient’s baseline QTc is above 500 ms, prescribing or continuing potentially QTc-prolonging medications may be relatively contraindicated. However, the risk-benefit of prescribing such medications is less clear when QTc is elevated (≥450 ms in males or ≥460 ms in females) but below 500 ms. Elucidating any dose-dependent relationships between possible QTc-prolonging drugs and relative QTc prolongation is important in determining safe drug dosing in high-risk patients. In this study, cumulative 24-hour doses of haloperidol ≥5 mg were not significantly associated with greater relative QTc prolongation than doses less than 5 mg. This may be due to the small sample size. Further research in this direction, with both haloperidol specifically as well as other medications, is needed.

Loop diuretics work by binding to and inhibiting the sodium-potassium-chloride symporter, reducing sodium, potassium, and chloride reabsorption [16]. This process increases diuresis and natriuresis but may also prolong QTc through electrolyte imbalances, particularly hypokalemia. In our study, diuretic use was associated with QTc ≥500 ms in unadjusted analysis. However, this finding should be interpreted cautiously because diuretic use may serve as a marker of underlying conditions such as heart failure and volume overload, which themselves may be associated with QTc abnormalities. The small number of patients with QTc ≥500 ms also precluded reliable multivariable adjustment.

Interestingly, serum potassium levels were not significantly different between patients with and without marked QTc prolongation, which suggests that there may be other side effects of loop diuretics that can affect the QT interval. Snitker et al. demonstrated that QTc prolongation can occur with furosemide in patients with chronic kidney disease, even after adjusting for serum potassium and calcium [17]. Intracellular electrolyte shifts, which may not be captured in serum chemistry studies, are one possible mechanism for this finding. In light of these findings, caution may be advised with the concurrent administration of loop diuretics and haloperidol, especially in patients already at high risk for QTc prolongation.

Serum magnesium concentrations were significantly higher among patients who developed QTc ≥500 ms. This finding was contrary to the expected association between hypomagnesemia and QTc prolongation and should therefore be interpreted cautiously. One possible explanation is that patients perceived to be at increased risk for arrhythmia may have undergone electrolyte replacement before measurement, such that serum magnesium at the time of QTc assessment may not reflect the patient’s earlier magnesium status. Alternatively, the finding may represent a chance association related to the small sample size and multiple comparisons.

This study has several important limitations. First, the sample size was relatively small, which limited statistical power and increased the likelihood of both false-positive and false-negative findings. Second, the study included only patients already classified as high risk by a modified Tisdale score. There was no low-risk or unflagged comparison group, and the study cannot assess the discrimination, calibration, sensitivity, specificity, or clinical effectiveness of the automated risk score. Third, the observational design and lack of a non-haloperidol comparison group prevent causal attribution of observed QTc changes specifically to haloperidol. Acute illness, baseline cardiac disease, electrolyte replacement, diuretic therapy, other QT-prolonging medications, and other unmeasured factors may have contributed to QTc changes. Fourth, associations were evaluated using univariate analyses because the small sample size and limited number of outcome events did not support reliable multivariable modeling. Finally, ECG and telemetry measurements were obtained at discrete time points and may not fully capture transient QTc changes.

## Conclusions

QTc prolongation was common among hospitalized patients identified as high risk for TdP who received haloperidol, with 50% experiencing a QTc increase of ≥20 ms and 25% developing QTc ≥500 ms. Diuretic use was associated with marked QTc prolongation in unadjusted analysis, although the small sample size and potential confounding preclude causal inference. These findings support further investigation of automated risk stratification as a means of identifying patients who may benefit from targeted QTc monitoring and medication review.
